# Sex-Specific Associations of Testosterone With Metabolic Traits

**DOI:** 10.3389/fendo.2019.00090

**Published:** 2019-03-13

**Authors:** Stefan Z. Lutz, Robert Wagner, Louise Fritsche, Andreas Peter, Ingo Rettig, Caroline Willmann, Ellen Fehlert, Peter Martus, Tilman Todenhöfer, Norbert Stefan, Andreas Fritsche, Hans-Ulrich Häring, Martin Heni

**Affiliations:** ^1^Division of Endocrinology, Diabetology, Vascular Disease, Nephrology and Clinical Chemistry, Department of Internal Medicine, University of Tübingen, Tübingen, Germany; ^2^Institute for Diabetes Research and Metabolic Diseases of the Helmholtz Center Munich at the University of Tübingen, Tübingen, Germany; ^3^German Center for Diabetes Research, Neuherberg, Germany; ^4^Institute of Clinical Epidemiology and applied Biostatistics, University of Tübingen, Tübingen, Germany; ^5^Department of Urology, University of Tübingen, Tübingen, Germany

**Keywords:** testosterone, insulin sensitivity, insulin secretion, hyperandrogenemia, hypogonadism

## Abstract

**Background:** Testosterone levels are differentially linked with diabetes risk in men and women: lower testosterone levels in men and higher testosterone levels in women are associated with type 2 diabetes, though, the mechanisms are not fully clear. We addressed sex-specific links between testosterone and major pathogenetic mechanisms of diabetes.

**Methods:** We analyzed data of 623 subjects (202 male, 345 female without, and 76 female with oral contraceptive therapy [OCT]) for whom insulin sensitivity and insulin secretion were assessed by oral glucose tolerance test. Body fat percentage was assessed by bioelectrical impedance. Testosterone was measured by enzyme-linked immunoassay; free testosterone and Framingham risk score were calculated.

**Results:** There were significant interactions between testosterone and sex for all tested metabolic traits. Increasing testosterone was associated with less body fat, elevated insulin sensitivity, and reduced glycemia, independent of adiposity in men. In women without OCT, testosterone correlated with more body fat, insulin resistance, and higher glucose concentrations. Testosterone was not associated with insulin secretion in either sex, but with lower Framingham risk score in men and higher Framingham risk score in women.

**Conclusions:** Similar to diabetes risk, insulin resistance has different association directions with testosterone levels in males and females. Insulin resistance could therefore constitute the best biological candidate linking testosterone levels and diabetes prevalence. The question of antiandrogen therapy being able to improve metabolism, glucose tolerance and cardiovascular risk in women was not clarified in our study but should be reviewed with higher numbers in a carefully matched study to reduce the influence of confounding variables.

## Introduction

Epidemiological studies observed a divergent association between testosterone levels and risk for type 2 diabetes in men and women: in men, lower testosterone levels are associated with elevated risk for type 2 diabetes. In contrast, higher testosterone levels associate with increased diabetes risk in women (1). At least in men, low testosterone is even associated with cardiovascular mortality (2).

Polycystic ovary syndrome (PCOS) is the condition that most often causes elevated testosterone concentration in women (3). Besides inducing clinical signs of hyperandrogenemia, PCOS is strongly associated with diabetes risk (4). In men, however, marked pharmacological lowering of circulating testosterone levels by androgen deprivation therapy, e.g., for the treatment of prostate cancer, increases diabetes risk (5). The detailed mechanisms underlying these two observations are still under investigation.

The most important pathogenic elements for type 2 diabetes are insulin resistance combined with the failure of pancreatic insulin secretion. When analyzing the sex-specific influence of testosterone on these two key factors, important confounding factors have to be taken into account. At least in men, testosterone concentrations decline with age (6) as well as with progressive obesity (7). A number of studies suggest an association between testosterone and either insulin sensitivity or insulin secretion [for overview see ref. (8–10)], though, no systematic investigations of these factors together with glucose metabolism in a large group have been published so far. We therefore carefully investigated the association of testosterone with glycemic traits such as insulin sensitivity and insulin secretion, with special focus on the existence of a sexual dimorphism in a deeply phenotyped cohort.

## Materials and Methods

### Patient's Characteristics

We analyzed data of 421 female and 202 male subjects from the ongoing TUEF study (Tuebingen Family Study), that includes persons at elevated risk for type 2 diabetes (family history of type 2 diabetes or overweight/obesity or previous diagnosis of impaired glucose tolerance or of gestational diabetes) (11), for whom both insulin sensitivity and insulin secretion were assessed during a frequent sampling 75 g oral glucose tolerance test (data closure: 2018-03-08). Patient's characteristics are presented in the Table 1. Female data were sorted on the basis of use of oral contraceptive therapy (OCT). Data cohort numbers were: male (*n* = 202), female without OCT (*n* = 345) and female with OCT (*n* = 76). Body fat percentage was assessed by bioelectrical impedance analysis using the BIA 101 analyzer from Akern bioresearch srl, Florence, Italy, and the software Cyprus Version 2.7 from RJL Systems, Clinton Township, MI, USA.

**Table 1:** Characteristics of the participants.

	**Men**	**Women without OCT**	**Women with OCT**
N	202	345	76
Age (yr)	38.4 [25–48.3]	37.7 [31–42.5]	30.1 [24–35]
BMI (kg/m^2^)	27.8 [22.8–28.8]	28.7 [22.2–32.8]	25.5 [21.6–27.1]
Waist-to-hip ratio	0.91 [0.85–0.96]	0.83 [0.79–0.88]	0.81 [0.75–0.85]
Body fat content (%)	21.4 [16.4–24.6]	36.7 [28–45.4]	32.3 [24.3–38.4]
Fasting glucose (mmol/l)	5.1 [4.7–5.4]	5.2 [4.7–5.4]	4.7 [4.5–5]
Glucose 120 min (mmol/l)	5.9 [4.5–6.7]	6.3 [5–7.1]	6.2 [5-7]
OGTT-derived insulin sensitivity index (AU)	16.6 [8.1–23.7]	14 [6.8–18.1]	17.2 [9–21.8]
C-peptide 0 min (pmol/l)	631.5 [447.8–759.3]	608.1 [397–711.8]	560.3 [421.8–702.5]
C-peptide 30 min (pmol/l)	2042.3 [1400.5–2430.5]	1825.8 [1338.5–2150]	1817.8 [1412–2176.5]
Total testosterone (nmol/l)	17.91 [12.77–22.47]	1.51 [1.07–1.87]	1.08 [0.61–1.52]

*Presented are medians [interquartile range]. OCT, oral contraceptive therapy*.

### Determination of Insulin Sensitivity

Insulin sensitivity was estimated from the OGTT as proposed by Matsuda and DeFronzo (12).

### Oral Glucose Tolerance Test (OGTT)-Derived Insulin Secretion

This was calculated as area under the curve (AUC) according to the trapezoid method as ½[½(C-Pep0′) + C-Pep 30′ + C-Pep60′ + C-Pep 90′ + ½(C-Pep_120_)]/½[½(Glc 0′) + Glc 30′ + Glc60′ + Glc 90′ + ½(Glc 120′)], with C-Pep = C-peptide.

### Laboratory Measurements

Serum testosterone was measured on ADVIA Centaur and sexual-hormone binding globulin (SHBG) on Immulite (Siemens Healthineers). The hormone measurements have been performed in a routine diagnostic laboratory under accreditation with the German accredited body (DAkkS). Internal and external quality control was performed at all times during the study including proficiency testing 4 times a year and passed at all times.

The ADVIA Centaur testosterone assay approved for the measurement of testosterone in men, women and infants designed to be comparable to an isotope dilution-liquid chromatography-tandem mass spectrometry (ID-LC-MS/MS) testosterone method (Centers for Disease Control and Prevention Hormone Standardization Program (CDC HoSt) Testosterone Reference Measurement Procedure). According to the manufacturer, a typical correlation coefficient for the comparison of the two methods is *r* = 0.98. The co-efficient of variation (CV%) for testosterone method was 4.5% at 15–25 nmol/L and 6% at 3.5 nmol/L. The assay shows a high specificity with no relevant cross-reactivity with other endogenous steroids.

Based on serum testosterone, SHBG and albumin levels, free testosterone in serum was calculated for women as previously reported (13). For most men, SHBG measurements were not available, thus free testosterone was not calculated for men.

### Estimation of Cardiovascular Risk

The Framingham risk score was calculated as previously reported (14). Missing values of systolic blood pressure were imputed using multivariate imputation by chained equations (https://www.jstatsoft.org/v045/i03, assessed February, 1st 2018).

### Statistical Analysis

Statistical analyses were conducted using JMP 13.0 or R version 3.4 (15). Associations of testosterone with insulin sensitivity, basal glucose and glucose 120 min were tested in multivariate linear regression analyses of log-transformed data with adjustment for age and body fat percentage, while associations with insulin secretion were tested after adjustment for age and insulin sensitivity. Exploratory, quadratic terms were included for testosterone. A *p* < 0.05 was considered to be statistically significant.

## Results

**Figure 1:**
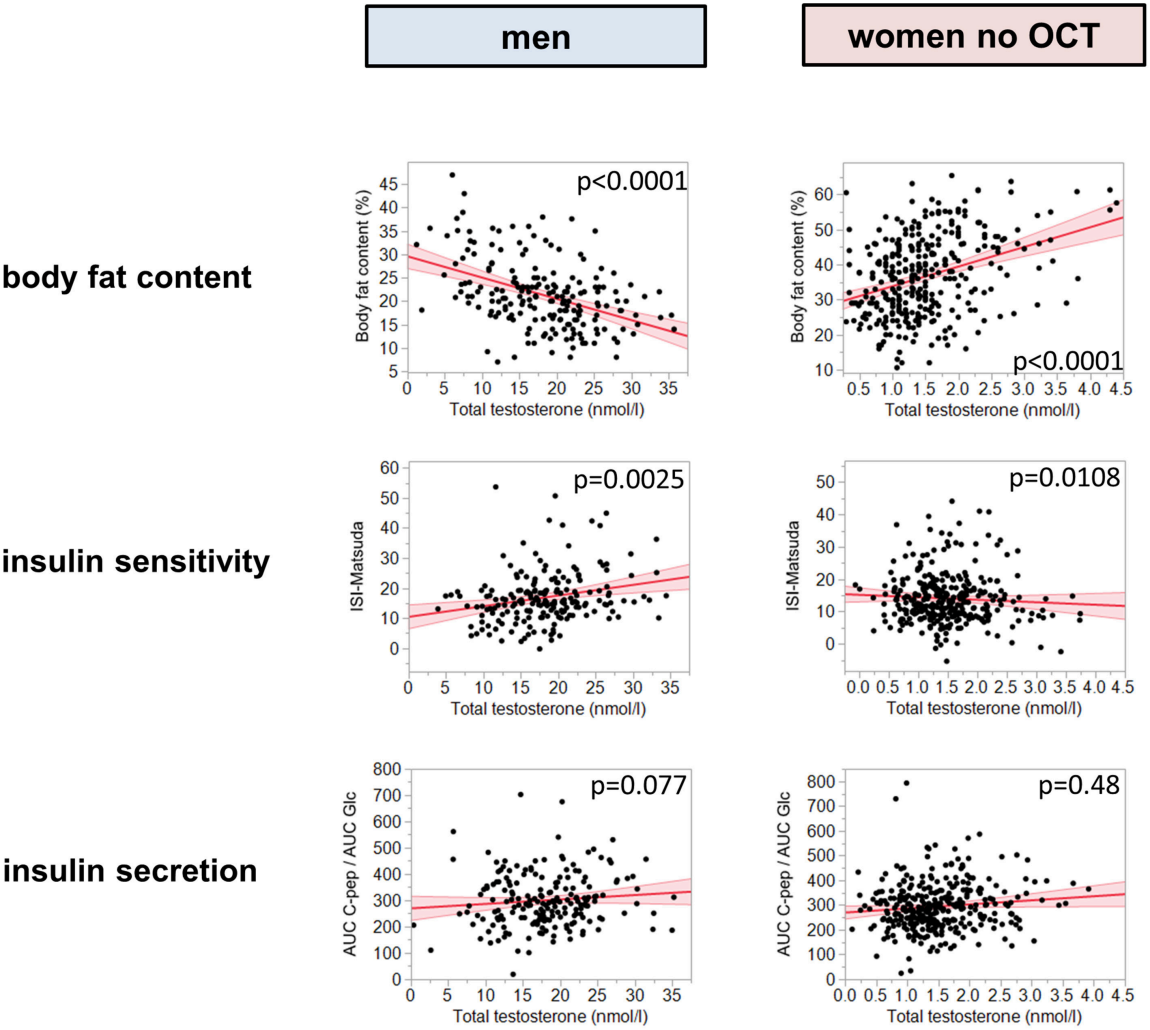
Presented are metabolic traits plotted against testosterone. The left column presents results for total testosterone in men, the right column presents total testosterone in women without OCT. The first line reports body fat content, the second insulin sensitivity, and the third insulin secretion, assessed as AUC C-peptide/AUC glucose. Red line represents fit line ±95% CI. Associations were tested by multiple linear regression analyses of log-transformed data. Insulin sensitivity was adjusted for body fat content and age, insulin secretion was adjusted for age and insulin sensitivity. OCT, oral contraceptive therapy.

There were significant interactions between total testosterone levels and sex for all tested metabolic traits (*p* ≤ 0.0015). Therefore, further analyses were conducted after stratification by sex. Furthermore, OCT intake in women significantly interacted with total testosterone on insulin sensitivity (*p* = 0.04). Total testosterone levels were negatively associated with body fat content in men (*p* < 0.0001, β = -0.40), but positively in women (*p* < 0.0001, β = 0.25, Figure 1; free testosterone: *p* = 0.0002, β = 0.34, Supplementary Figure 1). There was again a significant interaction between OCT intake and testosterone in women (*p* = 0.015). After adjustment for age and body fat percentage, higher total testosterone levels were associated with elevated insulin sensitivity in men (*p* = 0.0025, β = 0.18, Figure 1 and Supplementary Table), but in contrast, with reduced insulin sensitivity in women without OCT (*p* = 0.011, β = -0.11, Figure 1 and Supplementary Table; free testosterone: *p* = 0.0002, β = −0.33, Supplementary Figure 1). Similar results were found using quadratic polynomial functions for total testosterone (Supplementary Figure 2). Total testosterone was not correlated with insulin secretion in both groups of women (*p* = 0.48, β = 0.04 without OCT, *p* = 0.63, β = 0.05 with OCT, Figure 1 and Supplementary Table; for free testosterone all *p* > 0.94) or in men (*p* = 0.077, β = 0.14, Figure 1 and Supplementary Table, interaction not significant). Total testosterone was negatively correlated with fasting glucose and post-challenge glucose at 120 min in men (*p* = 0.001, β = −0.24 and *p* = 0.004, β = −0.21, respectively), while in women without OCT, free testosterone was positively associated with both glycemic parameters (*p* = 0.031, β = 0.23 and *p* = 0.004, β = 0.28, respectively). The association with fasting glucose did not reach statistical significance for total testosterone in women without OCT (*p* = 0.79, β = 0.02), but post challenge glucose at 120 min was also positively correlated with total testosterone (*p* = 0.001, β = 0.19).

In contrast to women who did not take OCT, no correlation between total or free testosterone and any of the tested metabolic traits was found in the group of women with OCT (*p* ≥ 0.14).

In addition, there was a significant interaction between testosterone and sex for the Framingham risk score (*p* < 0.0001). In men, total testosterone was negatively associated with the Framingham risk score (*p* = 0.0003). The association between free testosterone and Framingham risk score was only significant for women who did not take OCT (*p* = 0.041).

## Discussion/Conclusions

This study found opposite associations for testosterone levels and metabolic traits between sexes (in non-OCT females): while testosterone was linked to less body fat, better insulin sensitivity, and lower glucose levels in men, it was linked to adiposity, insulin resistance, and higher glucose concentrations in women. Nevertheless, in women taking OCT no statistically significant associations were detected.

Our data suggest that insulin resistance could be the link between the sex-specific association of testosterone and type 2 diabetes risk. This is explained only in part by increased body adiposity, as there was an association of testosterone with insulin resistance independent of body fat content. In previous studies in women with polycystic ovary syndrome, high testosterone levels were reported to stimulate insulin secretion [for overview see ref. (8)]. We could not replicate this interaction, possibly due to differences in study populations or because we accounted for possible confounding factors like age, body weight and OCT intake.

At least in male rodents, presence of the androgen receptor has been demonstrated in pancreatic β-cells, and knock-out of this receptor impaired insulin secretion. Our data suggest a weak link between testosterone and β-cell function in men; however, this did not reach statistical significance (16).

These sex-specific associations appear not only to be relevant for metabolism, but also for cardiovascular risk. While testosterone was linked to lower cardiovascular risk in men, it correlated with higher cardiovascular risk in women, as assessed by the Framingham risk score. Whether this relationship is explained by the known cardiovascular risk factor insulin resistance, needs to be investigated.

Since OCT use leads to a reduction of gonadotropins, it also modulates gonadotropin-dependent testosterone levels. The absences of an association of testosterone and insulin sensitivity in OCT users point to a causal role of sex hormones in modulating insulin sensitivity. Of note, accumulating evidence indicates a strong sexual dimorphism in brain processes that modulate peripheral metabolism and could therefore contribute to the current findings (17). Obviously, this hypothesis has to be tested in experimental studies, which specifically investigate the involvement of brain activity in a prospective setting. Further investigations should also test free testosterone using the gold standard mass spectrometry methodology, which was unfortunately not available in the current project. Another limitation of our current analysis is the smaller sample size of women taking OCT. However, the interaction between OCT intake and testosterone detected for insulin sensitivity and body adiposity argues for a true impact of OCT and against a sole problem of statistical power.

Our current results may help to partially explain the metabolic benefits of testosterone replacement in men with hypogonadism. We hypothesize that treating women who suffer from hyperandrogenemia with antiandrogen OCT might not only improve clinical features like hirsutism, but may have additional benefits on metabolism, glucose tolerance, and cardiovascular risk.

## Ethics Statement

This study was carried out in accordance with the recommendations of the Ethics Committee of the University of Tübingen with written informed consent from all subjects. All subjects gave written informed consent in accordance with the Declaration of Helsinki. The protocol was approved by the Ethics Committee of the University of Tübingen.

## Author Contributions

SZL and MH designed the study, performed analyses, and drafted the manuscript. LF, AP, and IR performed laboratory measurements and contributed to discussion. PM performed statistical analyses. TT contributed to discussion. RW, CW, EF, and NS examined study participants and contributed to discussion. AF and HUH supervised the project and contributed to discussion. All authors approved the final version of the manuscript prior to submission.

### Conflict of Interest Statement

The authors declare that the research was conducted in the absence of any commercial or financial relationships that could be construed as a potential conflict of interest.

## Abbreviations

CVD: cardiovascular disease
SHBG: sexual-hormone binding globulin
OCT: oral contraceptive therapy
OGTT: oral glucose tolerance test.
