# Supplementary material for: Sex-Specific Associations of Testosterone With Metabolic Traits
Source: Front Endocrinol (Lausanne). 2019 Mar 13;10:90. doi: 10.3389/fendo.2019.00090 (PMC6425082; doi:10.3389/fendo.2019.00090)
Supplement: Supplementary file 1 [file Table_1.docx]

**Supplementary table. Multivariate models.** Presented are associations of total testosterone in men and women with insulin sensitivity and insulin secretion. *log2-transformed.

1. **Association of total testosterone with insulin sensitivity in multivariable models in males**

| R^2^=0.48 | beta | p-value |
| --- | --- | --- |
| Age* | β=-0.317 | p<0.0001 |
| Body fat content* | β=-0.462 | p<0.0001 |
| Total testosterone* | β=0.183 | p=0.0025 |

1. **Association of total testosterone with insulin sensitivity in multivariable models in females without OCT**

| R^2^=0.42 | beta | p-value |
| --- | --- | --- |
| Age* | β=-0.038 | p=0.382 |
| Body fat content* | β=-0.611 | p<0.0001 |
| Total testosterone* | β=-0.115 | p=0.011 |

1. **Association of total testosterone with insulin sensitivity in multivariable models in females with OCT**

| R^2^=0.40 | beta | p-value |
| --- | --- | --- |
| Age* | β=-0.036 | p=0.692 |
| Body fat content* | β=-0.644 | p<0.0001 |
| Total testosterone* | β=-0.056 | p=0.534 |

1. **Association of total testosterone with insulin secretion in multivariable models in males**

| R^2^=0.09 | beta | p-value |
| --- | --- | --- |
| Age* | β=-0.237 | p=0.003 |
| Insulin sensitivity* | β=-0.365 | p<0.0001 |
| Total testosterone* | β=0.141 | p=0.077 |

1. **Association of total testosterone with insulin secretion in multivariable models in in females without OCT**

| R^2^=0.08 | beta | p-value |
| --- | --- | --- |
| Age* | β=-0.067 | p=0.213 |
| Insulin sensitivity* | β=-0.286 | p<0.0001 |
| Total testosterone* | β=0.039 | p=0.482 |

1. **Association of total testosterone with insulin sensitivity in multivariable models in females with OCT**

| R^2^=0.15 | beta | p-value |
| --- | --- | --- |
| Age* | β=-0.423 | p=0.0002 |
| Insulin sensitivity* | β=-0.145 | p=0.182 |
| Total testosterone* | β=0.051 | p=0.635 |

| R^2^=0.69 | beta | p-value |
| --- | --- | --- |
| Age* | β=0.842 | p<0.0001 |
| Body fat content* | β=-0.008 | p=0.861 |
| Total testosterone* | β=0.034 | p=0.458 |

1. **Association of total testosterone with Framingham Risk Score in multivariable models in males**
2. **Association of total testosterone with Framingham Risk Score in multivariable models in females without OCT**

| R^2^=0.49 | beta | p-value |
| --- | --- | --- |
| Age* | β=0.665 | p<0.0001 |
| Body fat content* | β=0.175 | p<0.0001 |
| Total testosterone* | β=0.033 | p=0.433 |

1. **Association of total testosterone with Framingham Risk Score in multivariable models in females with OCT**

| R^2^=0.55 | beta | p-value |
| --- | --- | --- |
| Age* | β=0.672 | p<0.0001 |
| Body fat content* | β=0.233 | p=0.004 |
| Total testosterone* | β=0.103 | p=0.191 |
