# Supplementary figures and images for: Sex-Specific Associations of Testosterone With Metabolic Traits
Source: Front Endocrinol (Lausanne). 2019 Mar 13;10:90. doi: 10.3389/fendo.2019.00090 (PMC6425082; doi:10.3389/fendo.2019.00090)

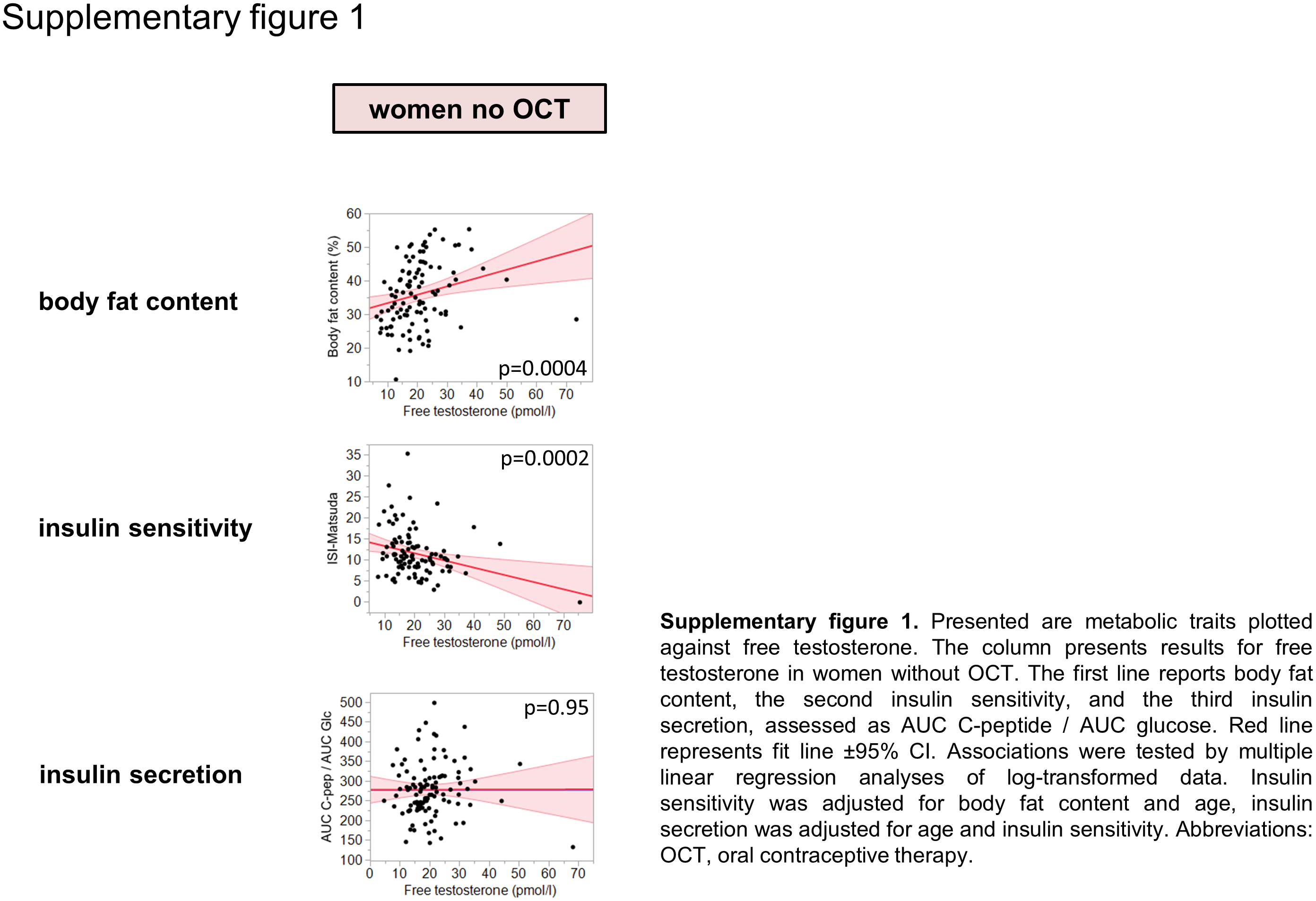

Supplement: Supplementary file 2 [file Image_1.TIF]

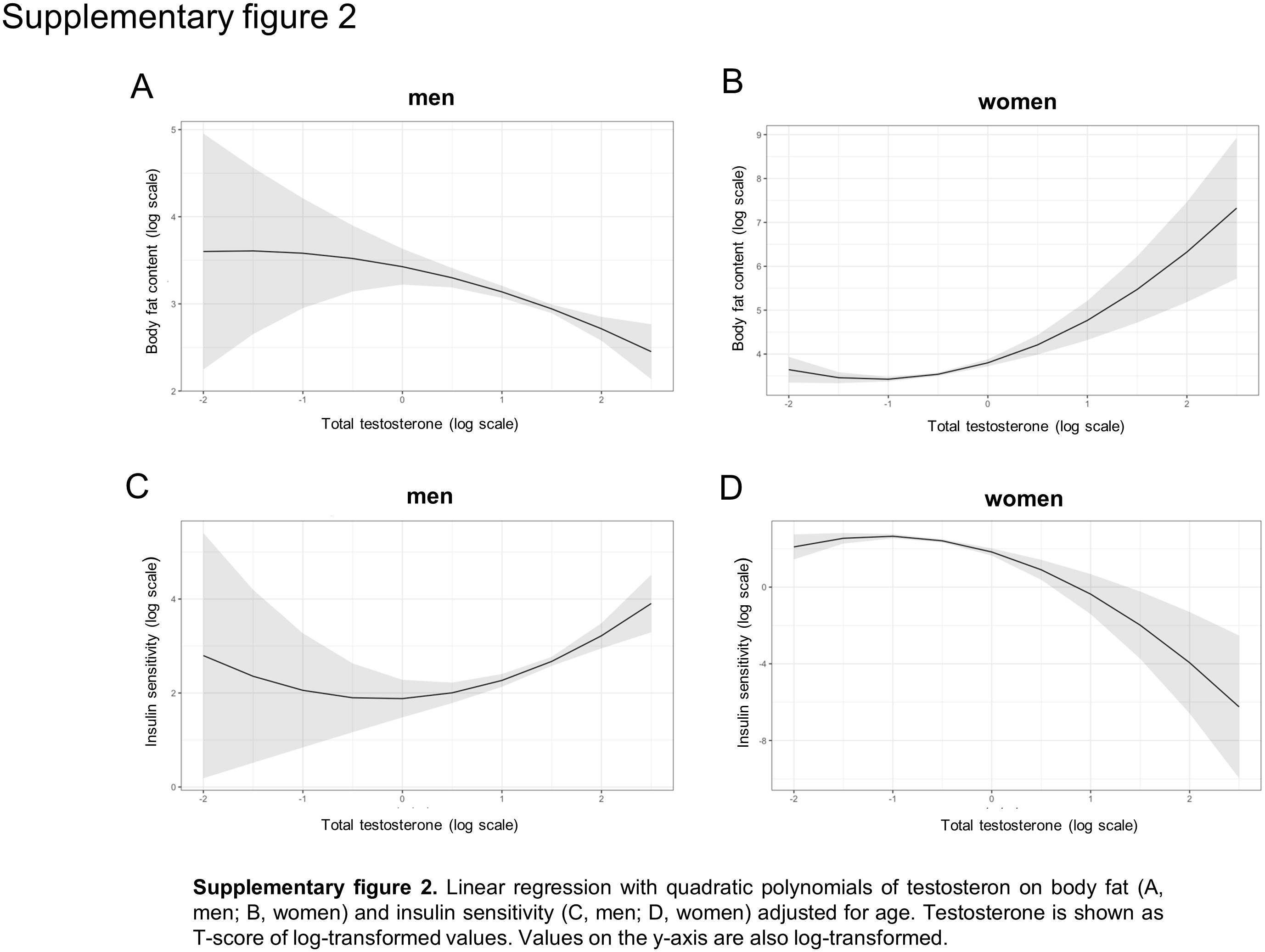

Supplement: Supplementary file 3 [file Image_2.TIF]
